# Gift-Giving and Network Structure in Rural China: Utilizing Long-Term Spontaneous Gift Records

**DOI:** 10.1371/journal.pone.0102104

**Published:** 2014-08-11

**Authors:** Xi Chen

**Affiliations:** School of Public Health, Institution for Social and Policy Studies, Department of Economics, Yale University, New Haven, Connecticut, United States of America; University of Kent, United Kingdom

## Abstract

The tradition of keeping written records of gift received during household ceremonies in many countries offers researchers an underutilized means of data collection for social network analysis. This paper first summarizes unique features of the gift record data that circumvent five prevailing sampling and measurement issues in the literature, and we discuss their advantages over existing studies at both the individual level and the dyadic link level using previous data sources. We then document our research project in rural China that implements a multiple wave census-type household survey and a long-term gift record collection. The pattern of gift-giving in major household social events and its recent escalation is analyzed. There are significantly positive correlations between gift network centrality and various forms of informal insurance. Finally, economic inequality and competitive marriage market are among the main demographic and socioeconomic determinants of the observed gift network structure.

## Introduction

Families in the world celebrate a variety of household social events, involving wedding, funeral, and childbirth and so on. Spontaneous written records for gift exchanges during ceremonies are widely available, such as in Thailand, Vietnam, China and Korea. The purpose is to record gifts received from hosting ceremonies to remind how much to pay back when fellow residents hold social events in the future. The widely preserved gift record tradition offers researchers a means with great potential to collect valuable datasets for social network analysis. Importantly, this data source possesses a number of unique features lacking in previous network datasets that well fit social network studies at both the individual level and the dyadic link level. To my knowledge, this data source has rarely been utilized in economics studies. Our paper attempts to draw researchers' attention to this much underutilized data source and analyze gift exchange patterns and gift networks structure in rural China.

The analysis is ordered into three parts. First, we summarize five unique features of gift records that improve social network analysis at different levels. We then introduce our project that matches a census-type longitudinal household survey from 26 villages in rural western China between 2004 and 2010 with a long-term household gift exchange data. One of the primary aims of the project was to gather quantitative data on the evolution of social networks to improve our understanding of how it shapes the network structure we observe and how it affects social behavior and individual well-being.

Second, the overall trend of household ceremonies and gift-giving in rural China is documented and major ceremonies are compared, emphasizing wider participation in some events relative to the others, lavish spending on social events and its recent escalation. Our results suggest that concern for relative standing due to worsening income inequality and competitive mating pressure, rather than avoiding social exclusion, offers more plausible explanation of the observed gift exchange pattern. Besides, the timing of gift spending escalation coincides with fast increase in windfall and other non-earned income, such as remittance, in rural China, which leaves open questions for future exploration. Note that remittances include all cash and in-kind goods sent back by migrants in one's own extended families, while gifts are exchanged with other families during ceremonies. Moreover, while informal risk-sharing through mutual assistance in labor, job information, production tools and informal credit may not be the cause of gift escalation, it is likely to play an important role in maintaining gift exchange ties.

Third, we further explore demographic and socioeconomic determinants of gift networks structure, which is evolved over long-term gift exchanges. Blood relatives' networks, economic status and economic inequality, age profile and marriage market pressure are found to affect major network structure indicators.

Besides accumulating knowledge of social networks and their associations with social behavior and well-being, the proposed method for social network data collection in this paper and insights on unique features of gift record data may shape future studies that overcome some of the important issues in the existing data. However, we realize that our way may hardly be general enough to apply to all other data collection efforts due to budget constraint, time constraint or local implementation issues.

We recognize that the size of gift exchanges in Guizhou province may not be nationally representative, but its preserved gifting norm in the past hundreds of years has resembled most other regions in China. The Chinese, rich and poor [7], living in eastern and western China [36], [38], rural and urban [29], and political elites and grassroot citizens [30], have been reciprocally presenting gifts during a very similar set of ceremonies and offering assistance in the long run to create, maintain and strengthen various interpersonal social ties and enhance the sense of belonging in the community. Comparing household expenses on wedding and funeral gifts in nine Chinese provinces, the expenditure for rural residents in Guizhou is smaller than the national average [38]. Meanwhile, the national average expenses on wedding gifts alone accounts for a substantial share of household income. Moreover, major ceremonies in rural Guizhou are documents and related to broader behavior and well-being issues that China faces [30]. All these evidence suggests the scientific generality of the method we use to collect gift receiving records.

The rest of the paper is organized as follows: Section 2 discusses sampling methods for existing social network studies and highlights the unique features of the gift record data. Section 3 describes the data collection process. Section 4 reports empirical results, including the trend of household ceremonies and gift-giving in rural China, correlations between gift-giving and various forms of informal insurance, and the socioeconomic determinants of network structure. Section 5 concludes.

## Methods for Social Network Sampling

The standard economics literature resorts to approaches founded on the principle of centralized human interaction in the market, anonymous agents, and uniform prices through which individuals coordinate [17]. For instance, the literature on social embeddedness measures social networks through membership in communities, ethnicity, gender, and geographic neighborhood and so on [12], [25]. However, differences in connections among heterogeneous agents (i.e. intensities, distributions and locations) through which social interactions flow, rather than memberships themselves, have an essential bearing on behavior [15], [24].

However, very few datasets have rich enough information on social network structure to get around the reflection problem in identifying the impact of social interactions [4], [23]. Studies that incorporate indirect social ties or more complex network relationships are scant [1], [2], and the snapshot feature of most network datasets determines that link and network dynamics are not well explored [13], [21]. Further, agent and link sampling biases as well as measurement errors hinder us from evaluating network impacts. Therefore, social network analysis calls for new datasets that enable researchers to understand the mechanism of social interactions via taking care of these key issues. This study collects complete records of gift exchanges, widely seen in social events linking people economically and socially but rarely studied in the literature.

### Features for Individual and Link Level Studies

One straightforward way to explore individual behavior in social networks is to sample individuals and collect social network information from them. There are three types of individual sampling: snowball sampling, random egocentric sampling, and census sampling. Snowball sampling starts from a set of initial respondents and enlarges the sample by adding in individuals mentioned by previous respondents. Sampling of relationships and individuals are simultaneously done. However, individuals following the first respondent are not randomly selected, which affects population inference [19]. Though most studies follow random sampling due to lower costs, it suffers from significant loss of information on network structure, total resources devoted to networks, and one's position in networks [33]. Complete network information on all agents' connections is desirable when we investigate individual network engagement [10]–[11], [16]. The gift records collected for all residents in each community provide such an example.

Sampling links is the immediate step following sampling individuals. Most studies include all the links (they could collect) among individuals in the sample (a.k.a. *matching within sample*), while a few randomly sample relationships (a.k.a. *random matching*). It is not apparent that one strategy is always superior to the other. From the perspective of preserving network information, *matching within sample* is more desirable when sampling ratio is high, which does not fit usual network datasets. *Random matching*, on the other hand, performs poorly when there is no restriction on the size of networks [26]. Fortunately, the gift records we collected obviate this flaw by preserving best possible features, i.e. high sampling ratio, unrestricted network size, and complete information on network structure.

Moreover, studies usually survey links at one point of time. The snapshot feature of the network data determines that the dynamics of links and networks are not well explored. Data of long-term gift-exchanges comes from five villages spreading between isolation and openness amid the fast growing Chinese economy, which enables us to explore network dynamics.

A common issue when studying at the dyadic link level is non-independent observations as a result of node-specific characteristics common to all links involving an agent. For example, agent A's gift to B can be correlated with A's gift to C as a result of generosity or budget constraint. Conventional OLS estimation generates consistent coefficient but inconsistent standard errors that bias towards finding an impact. Fortunately, a few approaches have been developed to correct the bias, such as the Quadratic Assignment Procedure [28], adjusting dyadic standard errors [13], multi-way clustering [6], and node fixed effects [11]. Two dyads containing no mutual members might still be correlated due to some common constraints. For example, gift given by agent A to B can be correlated to gift from C to D when both A and C are faced with budget constraint and therefore turn to individual E for help. To address this possibility, we can further cluster the observations by timing of the events. However, the snapshot feature of most previous network datasets makes it difficult to address this potential link dependence issue.

### Sampling and Measurement Error Corrections

First, most studies in the literature are not able to track ties with external agents [11], [13] due to massive costs. Therefore, inference from sample to population might be with error when significant non-random differences between identified links and missing links exist. This is probable, as there is, for example, less covariant shocks and therefore stronger motives of cross-community insurance. Moreover, isolated agents in a community might actually be linked to more agents out of sample. In all these situations, misleading results can be drawn [31]. Our gift record data, however, provides a rare case in which cross-community links are also captured. All identified gift senders and receivers are matched with our multi-wave household survey.

Second, when limiting the maximum links to be identified by each respondent (e.g. the Add Health Data), there is a risk of implicitly ranking relationships. Sometimes the “maximum number” strategy is difficult to implement when respondents refuse to rank people they regard as equally close to them. Meanwhile, it is possible that the order in which names are listed do not reflect their importance. Therefore, outcomes highly depend on specific contexts of the surveys and might be unable to replicate. Even if we aim to collect complete relationships, stronger ties usually have higher probability to be identified, while weaker ties are usually left out. This can be a problem when we explore information networks than informal insurance networks as *weak ties* matter more in the former case [18]. Fortunately, the gift record data imposes no maximum number of links. Moreover, it offers very objective information on the strength of relationships via the size of each gift.

Third, both real links [10], [22], [9], [31] and potential links [11], [13], [27] are used in the literature. The real relationships are largely recalled that may suffer from recall error, while gift records have no such error. In many countries with gift exchange tradition, all gifts are spontaneously recorded on the day of ceremonies with host families on site counting and checking them. If hosts are illiterates, their educated relatives help record each gift received. Therefore, gift records have no selection bias due to literacy.

Moreover, many surveys on networks do not explicitly distinguish receiving gifts/loans/help from giving [11]. To the surveys that can separate the two [13], [27], their short timeframe often makes it difficult to separate relationships involving only receiving or giving from those involving both giving and receiving. In contrast, our gift records are directional and in much longer term.

Further, self-reported networks can be biased to a large degree. For instance, respondents may take strategic actions when individual gift spending is elicited. Specifically, the poor might over-report gift expenditure to complain their huge social burden, while the rich may tend to hide their wealth by under-reporting gift expenditure. The opposite may be true when the poor treat enumerators as officials, thus under-report gift spending to compete for official subsidies. The rich may prefer to over-report to show social status. Therefore, some studies rely on proxy-report network information. For example, the above biases is expected to be substantially reduced when we elicit the same information from gift receivers. However, proxy-reported errors are often correlated with respondents' attributes or behavior due to projection bias. For instance, respondents in an informal insurance network may have more precise information on their closer fellow residents than more distant residents. As another example, innovative technology adopters tend to over-report the incidence of adoption in their networks. Thus, self-reported behavior and proxy-reported behavior are systematically different, but none of them may reflect the actual behavior [20]. Fortunately, spontaneous gift books kept by all gift receivers allow us to make use of the most reliable source of proxy-report network data, simultaneously avoiding errors in both self-report and proxy-report.

## Data Collection

Despite the ubiquity of gift giving in daily life, there is surprisingly little empirical evidence on the patterns of social spending across ceremonies and over time in Chinese communities, in large part due to lack of reliable data. To fill this gap, a primary household survey data collected from five randomly selected villages in Guizhou, China was matched to a spontaneous gift record data. The five villages represent the median level of the province. Like many other regions in rural western China, the mountainous landform keeps the five villages relatively isolated from the external communities. Among them, two villages are 10 kilometers away from the county seat with poor road access. In contrast, two village are only 2.5 kilometers away from the county seat. A high proportion of local residents migrate out, and shocks to their remittance may have large impact on gift and ceremony spending. In between, one village is populated with Buyi ethnic minority, who preserve culture differently from the other four Han villages. Ceremonies in this Buyi village are less costly, local residents usually participate in the events without bearing large burden on gift exchange. Overall, more than 20 ethnic groups live in the area, including Han, Miao, Buyi, Gelao, and Yi. In total, ethnic minorities comprise about 20% of population. Since the five villages are populated with Han group and ethnic minorities, we are able to explore social connections between ethnic groups.

All the participants in this study provided their written informed consent to participate in this study. The parents/guardians of the minors also provided their written informed consent to take part in this study and for their data to be used for research purposes. The survey design, implementation, and the result estimations of this study were all finished when the author was pursuing his Ph.D. degree at Cornell University. This manuscript was prepared as Chapter two of the author's dissertation at Cornell University. No new research using the data set has been conducted after coming to Yale University except polishing the wording of this chapter for journal publication. Therefore, only the IRB for Human Participants in the Office of Research Integrity and Assurance at Cornell University examined the project for potential ethics issues and approved the whole study, including methods and consent procedure for all participants. All the data used in this study has been anonymized.

First, upon verifying the availability and ruling out potential selection bias of gift record books during our pilot survey in August 2009, in 2010 we used digital cameras to collect gift book information for all major social occasions (including wedding, funeral, coming-of-age ceremony, child birth ceremony, and house-moving ceremony) hosted by all households (see Table 1 for main summary statistics). Gift-receiving records are usually kept by all households for a long time in order to pay back accordingly when other families hold celebrations. Ritualized gift giving is also associated with the custom of making and preserving gift lists. Gift lists are homemade books on red paper (funeral gift lists are made on yellow paper) inscribed with a traditional Chinese calligraphy brush. They serve as formal records of all gifts received by the host of a family ceremony. The gift books list the size of each gift received, the name of each gift presenter, the village where one resides, and whether the gift has been paid back [36].

**Table 1:** Summary Statistics (at the end of 2009).

		Mean	S.D.
Income inequality (Gini)	-	55.2	0.07
Normalized in-degree centrality (popularity)	-	0.04	0.22
Normalized out-degree centrality (Influence)	-	0.04	0.06
Normalized closeness centrality	-	0.34	0.05
Normalized Bonacich centrality	-	7.56	15.60
Income per capita (CNY)	-	2795.71	3716.14
Distance to the county seat (km)	-	5.58	3.25
Per capita cultivated land (mu)	-	1.05	1.05
Male head of household (dummy)	-	0.93	0.26
Education of household head (year)	-	4.15	3.20
Minority head of household (dummy)	-	0.32	0.47
Share of household members aged 11–29, unmarried (%)	-	0.21	0.23
Share of household members aged 60 and above (%)	-	0.13	0.27
Village leader or party member (Y/N)	-	0.09	0.29
Household head age (year)	-	47.12	12.81
# household members	-	3.81	1.64
Own farm machine (Y/N)	-	0.01	0.11
Own cow (Y/N)	-	0.24	0.65
Own horse (Y/N)	-	0.03	0.23
# occurance of big disease among family members in last two years	-	0.46	0.68
# occurance of livestock deaths in last two years	-	0.38	0.60

*Source:* Authors' survey data for five out of 26 villages where we collected gift records. N = 184.

The centerpiece is gift link identification. In contrast to recruiting a whole class of college students in our household survey, the field work to collect gift records were accomplished in a few months by my assistant and I who made continuous efforts to match the nicknames on the records with real names. We stayed in the villages and brought the roster for the villages to visit each household to identify those names not identifiable. The roster also made it easier for us to identify people who did not send gift to each family I visited and their relationships with the families, which are very important to the understanding of network formation. We also joined or hosted local public meetings to identify some hard nicknames. We identified all 184 households from the five villages with 8074 gift links that are within and between these villages, during 2005–2009. We also collected all available gift records for ceremonies between 1994 and 2004. Besides, 4611 cross-county/township gift links were recorded but not used in this study. Nearly all households' gift-receiving records for the ceremonies between 2005 and 2009 were included in this study except that one household reported loss of a wedding gift book. We consulted village leaders and local residents on major ceremonies before going to individual families. Besides, in our multi-wave household survey, we asked each respondent to recall all ceremonies they held in the last ten years. This prior information helped each household find gift books for us and aid us in verifying whether we collected gift books for all events.

**Figure 1:**
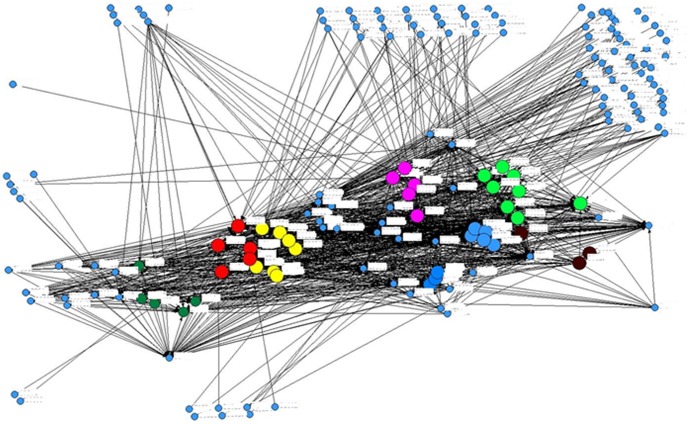
The Clan System and Gift Exchange Network in One of the Five Villages. *Source:* Author's social network data. *Notes:* Dots to the boundaries show households from other villages. Those bigger dots of the same color show households in the same clan. Households in the same clan usually live close to each other (as a result of the land inherited from their common ancestors) with intense gift exchanges.

Information on kinship and relatedness among villagers was also collected and matched to each gift link. As many other traditional rural communities, the five villages are all organized by long-term coordination of major clans. Figure 1 takes one of the five villages as an example.

Second, a three-wave (2005, 2007, 2010) census-type household survey was administered just before the most important Chinese spring festival when nearly all households (including those who worked outside their home villages during the year) were back home. The survey collected detailed information on villages, household demographics, income, consumption, gift expenditure and income, social events (e.g. weddings, funerals, and coming-of-age ceremonies) hosted during the past decade.

## Results and Discussion

### The Overall Trend of Ceremony and Gift-giving in Rural China

We first document the overall gift-giving pattern in rural China where the ceremony tradition and gift connections are preserved. The rich gift data, combined with longitudinal household survey, provide us a chance to look into gift spending and its recent escalation.

Table 2 presents average gift size per occasion (after adjusting for inflation), average number of households attending each social event, and average number of events per year in a community. Most ceremonies have witnessed tremendous increase in gift spending per event, which is supported by our three-wave survey in which respondents were asked to recall their average gifts to direct relatives, friends, and neighbors during major ceremonies between 2001 and 2009. After adjusting for inflation, the median gift per occasion jumped from 32 RMB to 77 RMB for direct relatives and from 31 RMB to 65 RMB for friends and neighbors. Meanwhile, all major ceremonies demonstrate booms in the number of guests attending each event and stable number of events each year in the communities, which suggest that total household gift expenditure has increased rapidly in recent years.

**Table 2:** Gift Spending and the Number and Size of Ceremonies (2000–2009, per Occasion).

Year	Coming-of-age			Male Wedding			Female Wedding			Funeral		
	Mean gift (CNY)	# events	Mean # guests	Mean gift (CNY)	# events	Mean # guests	Mean gift (CNY)	# events	Mean # guests	Mean gift (CNY)	# events	Mean # guests
2000–2004	28.8	2.2	35.5	41.7	0.65	31.0	41.6	0.53	22.1	23.5	2.19	31.1
2005	25.1	3.2	34.2	45.9	2.47	38.1	59.9	1.77	27.4	28.7	3.03	49.5
2006	27.6	4.2	41.3	55.4	1.94	34.3	58.1	0.97	31.0	21.8	3.13	61.9
2007	46.6	4.0	46.1	60.5	2.06	40.0	53.3	0.94	26.3	54.7	4.30	46.2
2008	61.8	3.3	37.6	73.6	2.75	35.5	59.7	1.13	36.2	85.4	3.32	56.0
2009	73.3	3.6	51.5	90.6	2.65	37.3	68.4	1.31	44.9	87.9	3.19	75.5

*Source:* Authors' gift exchange data from five villages.

*Notes:* CNY = yuan renminbi (Chinese currency).

[1] The gift books record all the gifts received and the corresponding names of gift givers in different occasions.

[2] The gift sizes have been adjusted to constant 2000 price (RMB) using the rural consumer price index published in *China Statistic Yearbook* (China National Bureau of Statistics, various issues).

**Figure 2:**
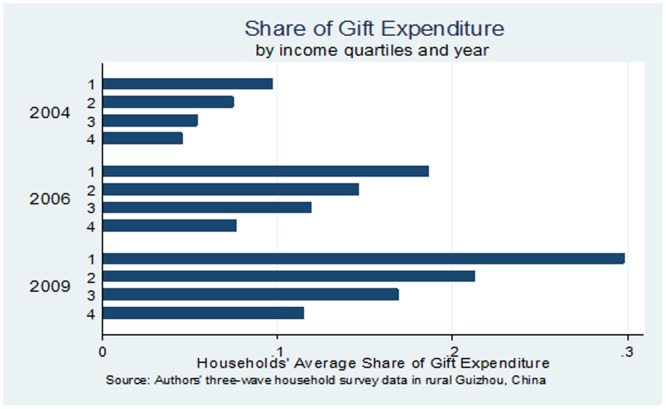
Income Share of Gift Expenditure. *Source:* Author's household survey data. *Notes: 1:* live with less than 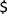
1 per day; 2: live with less than 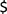
2 per day; 3: live with 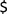
2–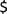
4 per day; 4: live with 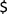
6–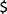
10 per day. The poverty lines are adjusted according to 2005 PPP rate from http://iresearch.worldbank.org/PovcalNet/jsp/index.jsp.

Figure 2 presents share of gift expenditure categorized by four income quartiles and year. The poorer a household is, the higher share of income is devoted to social spending, and the higher growth rate of gift expenditure share is between 2004 and 2009. Our results are consistent with Brown et al. [5] that gift spending in recent years far exceeds annualized growth in per capita income and other consumption. The increasingly large proportion of resources devoted to gifts and festivals spending, especially in impoverished areas, may have negative impact on well-being [8].

From organizers' perspective (Table 3), though gift-giving is recognized as an informal insurance against lumpy ceremony expenditures incurred, total expenditures in ceremonies amount to several times of their per capita income, especially weddings for grooms' families. The average gift size per occasion and average number of households attending each event in Table 2 indicate that gift income for an average event is far from enough to cover the expenses. Considering that gift spending increases rapidly, organizing an event today suggests even larger burden in the future to pay back when others hold ceremonies.

**Table 3:** Median Expenditures (RMB) in Major Ceremonies (1996–2009).

year	Coming-of-age	Wedding (Groom's Family)	Wedding Ceremony (Bride's Family)	Funeral expenditure
1996	-	4500 (**3.00**)	3157 (**2.10**)	2688 (**1.79**)
1997	-	3852 (**2.84**)	3100 (**2.29**)	3471 (**2.56**)
1998	-	5211 (**3.85**)	3025 (**2.23**)	3170 (**2.34**)
1999	-	3634 (**2.64**)	3829 (**2.79**)	4328 (**3.15**)
2000	-	6250 (**4.85**)	2929 (**2.27**)	4393 (**3.41**)
2001	-	7371 (**5.81**)	5644 (**4.45**)	3388 (**2.67**)
2002	-	7347 (**5.20**)	4536 (**3.21**)	3402 (**2.41**)
2003	-	7891 (**6.22**)	5143 (**4.05**)	4655 (**3.67**)
2004	-	10423 (**8.24**)	4243 (**3.35**)	6150 (**4.86**)
2005	3208 (**1.95**)	9486 (**5.76**)	7633 (**4.63**)	5156 (**3.13**)
2006	3387 (**2.62**)	11805 (**9.14**)	7502 (**5.81**)	6175 (**4.78**)
2007	4284 (**2.75**)	8569 (**5.50**)	4927 (**3.16**)	8096 (**5.20**)
2008	8046 (**5.50**)	13983 (**9.56**)	5833 (**3.99**)	7561 (**5.17**)
2009	8154 (**5.51**)	15066 (**10.18**)	7766 (**5.25**)	7151 (**4.83**)

*Source:* Authors' survey data.

*Notes:* Using Recall data from the 2007 survey and 2009 survey.

[1] All spending have been adjusted for inflation based on *China Statistic Year Book* published by NBS. All values are in RMB. [2] Recall data on organizing coming-of-age ceremony were only collected since 2005. [3] Numbers in brackets denote expenditure as times of per capita in.

### The Recent Gift Spending Escalation

We focus on reciprocal ties to measure the rate of gift escalation by ceremony. Since different ceremonies follow different market prices, reciprocal ties are defined as gift exchanges between a pair of households within the same category of events during 2000–2009. In our gift records, reciprocal ties are scant for time gap larger than six years, meaning that most of the gifts already paid back were in six-year timeframe.

**Figure 3:**
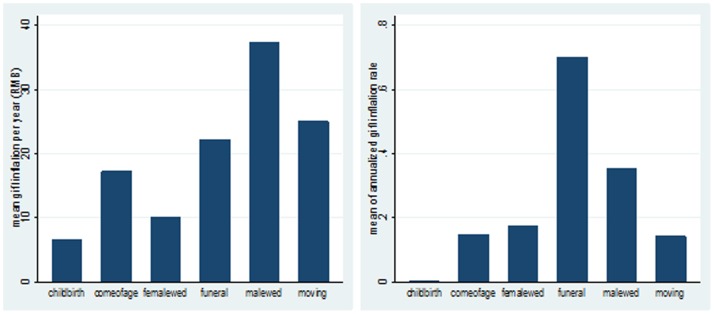
Annualized Mean Gift Exchange Inflation (RMB) and Inflation Rate for Reciprocal Households (2000–2009). *Source:* Author's social network data. *Notes:* All gifts have been adjusted for inflation based on *China Statistic Year Book* published by NBS. The left figure is **i**n terms of mean gift inflation per year (RMB), while the right figure is in terms of annualized gift inflation rate (100%).

Figure 3 presents gift inflation rates after adjusting for inflation. In terms of mean gift escalation per year (RMB), male wedding ranks the highest, followed by house-moving and funeral. In terms of annualized gift inflation rate, funeral ranks the highest, followed by male wedding. We also examine the relationship between time gap (gift repaying - gift receiving) and gift inflation for each ceremony, and the results are available upon request. To summarize, gift escalation (in terms of RMB and inflation rate) is the highest when a gift is paid back shortly afterwards, while it reduces as the time gap increases. Gifts in funerals have the highest immidiate inflation rate, which is followed by male weddings. The annualized gift inflation vanishes faster for funerals and male weddings than other ceremonies.

Why does gift spending increase rapidly? One hypothesis is that gift giving may serve as a social exclusion strategy. On the one hand, the larger the risk sharing network, the better the group may diversify their income risks; on the other hand, if the group includes a large proportion of households with persistently low income, the high income households may prefer to exclude them. However, the transition matrixes based on income information from the three-wave household survey do not reflect much rigidity in the five villages (Table S1 and Table S2). Specifically, even households in the bottom can climb the income ladder to rank higher. Meanwhile, Table 4 summarizes changes in network participation and activeness. Very few active households drop out of gift exchange activity or become less active, while their inactive counterparts significantly join or become more active in the networks. This pattern seems to be against the social exclusion hypothesis.

**Table 4:** Numbers of Households Join/Drop Out of the Gift-Exchange Network.

Panels	hhs (base year)	Active hhs (base year)	Among them: Active hhs (end year)	inactive hhs (base year)	Among them: inactive hhs (end year)
*Definition for “Active”:* centrality>0					
04–06	184	121	118	63	27
06–09	184	154	152	30	12

Panels	hhs (base year)	Very active hhs (base year)	Among them: very active hhs (end year)	Not very active hhs (base year)	Among them: not very active hhs (end year)
*Definition for “Very Active”: household centrality>mean(centrality) in the base year*					
04–06	184	88	85	96	49
06–09	184	132	121	52	33

(Households outside the five villages are excluded).

Source: Authors' gift exchange data from five villages.

*Notes:* The Bonacich centrality measure is normalized in UCINET 6. Households outside the five villages where we collected gift records are excluded as we only have partially information on their network links, which preclude us to calculate their network centralities.

An alternative hypothesis is the concern for relative standing, which can be motivated by income inequality as well as low rank in the pressured marriage market. China has experienced fast economic growth with worsening inequality. The average per capita income (adjusted for inflation) in surveyed communities has increased from 1404 CNY in 2004 to 2796 CNY in 2009, while the recent Gini coefficient is larger than 0.5 (Table 1). The enlarged income gap may motive people to climb social ladders. The tightening marriage market favoring brides may motivate households with son to engage in social events and signal wealth (via gift-giving) to matchmakers and bride families to increase the chance of getting married [5], [34].

It is well recognized that windfall and other non-earned income is often spent differently from earned income [7]. The recent fast growth in gift spending might be due to more opportunities to get access to lumpy windfall income or other non-earned income. For example, we find significant positive correlation between resettlement subsidy (targeting dilapidated houses and vulnerable habitats), land acquisitions subsidy (targeting villages close to the county seat affected by urbanization development program) and gift spending. Moreover, remittance from migrated family members may intensify gift spending. Table 2 shows booming gift spending in most ceremonies after 2005–2006, while Table 3 shows that ceremony organization has become more costly since 2004–2005. This trend coincides with the fact that China passed the Lewis turning point of unlimited labor supply after 2003 [37]. The passage of the turning point means significant rising wages in the labor market and potentially higher remittance. Apart from large windfall income and remittance, small official subsidies widely distributed over the past few years may also contribute to the escalating gift spending. For instance, direct grain subsidy was implemented in 2005, it targets grain growing area rather than yield. However, the causal impact of different sources of income on gift spending escalation deserves future study.

### In-kind Versus Cash Gift

Exchanging in-kind gifts during ceremonies has been a tradition. In wedding ceremonies, apart from large cash gifts, people send dumplings, pork, wool, woolen blankets, bed sheets, quilts, kitchen supplies, candles, wine, basins and pillows to the new couple to symbolize a sweet life or to help purchase necessities. During funeral ceremonies, people send less cash but more in-kind gifts and non-cash help. The in-kind gifts include corn, lamb, pork, woolen blankets, quilts, edible oil, wine as well as other sacrificial offerings. In celebrating coming-of-age occasion, people send rice and children's wear, while in child birth ceremonies people additionally give wool, eggs and fruits. When friends and relatives move their houses, furniture, stoves and curtain are usually sent as gifts.

In-kind gifts are widely seen in the following conditions: one, in impoverished villages residents send in-kind gift to avoid cash shortage; two, in some household celebrations saliently featured by reciprocal assistance, such as funeral and house-moving, in-kind gift indicates closeness between gift sender and receiver in addition to large amount of cash gift; three, in-kind goods are scarce and more attractive in remote areas with poor market access.

**Figure 4:**
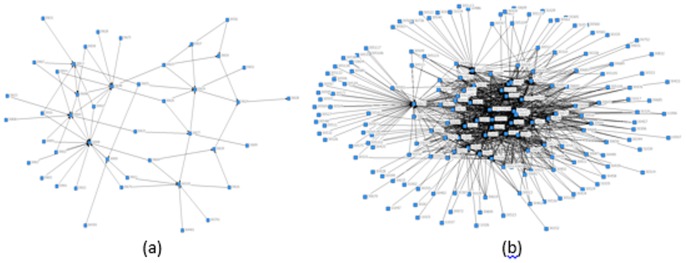
In-kind and Cash Gift Network in One of the Five Villages. *Source:* Author's social network data. *Notes:* (a) shows in-kind gift network, and (b) shows cash gift network.

However, cash gifts are more intense relative to in-kind gifts (Figure 4). While evidence from the western society demonstrates that sending cash to friends is associated with stigma [32], the pattern in rural China is the opposite that people tend to measure closeness by the size of cash gifts, motivating more cash gifts to substitute in-kind gifts. The contrasting pattern for cash and in-kind gift is more salient for more open villages.

### The Trend by Ceremonies

We introduce two key network concepts and measures in our comparison of gift-giving trend between ceremonies. *In-degree centrality*, a.k.a. *popularity*, measures direct links that one receives from peers, while *out-degree centrality*, a.k.a. *influence*, measures direct links that one sends out to peers. Both *out-degree centrality* and *in-degree centrality* are measured to capture density and distribution of gift links (see Table S3 for their measures) [14]. Overall, network centralities for both gift-sending (out-degree) and gift-receiving (in-degree) have been decreasing. This trend suggests more frequent and evenly distributed gift exchanges overtime. Based on resemblance for each pair of ceremonies, we make three comparisons.

**Figure 5:**
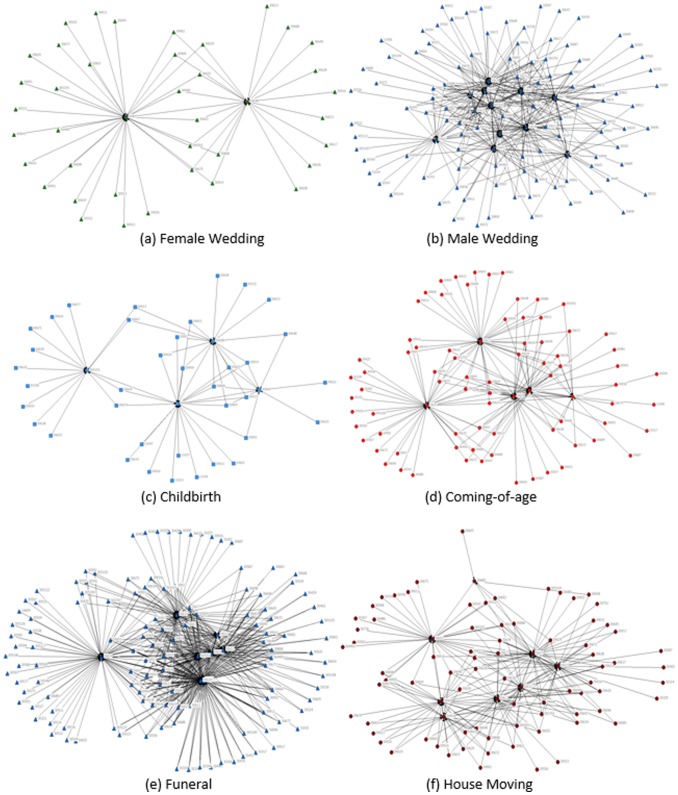
Gift Networks for Major Social Occasions in One of the Five Villages. *Source:* Author's social network data.

**Figure 6:**
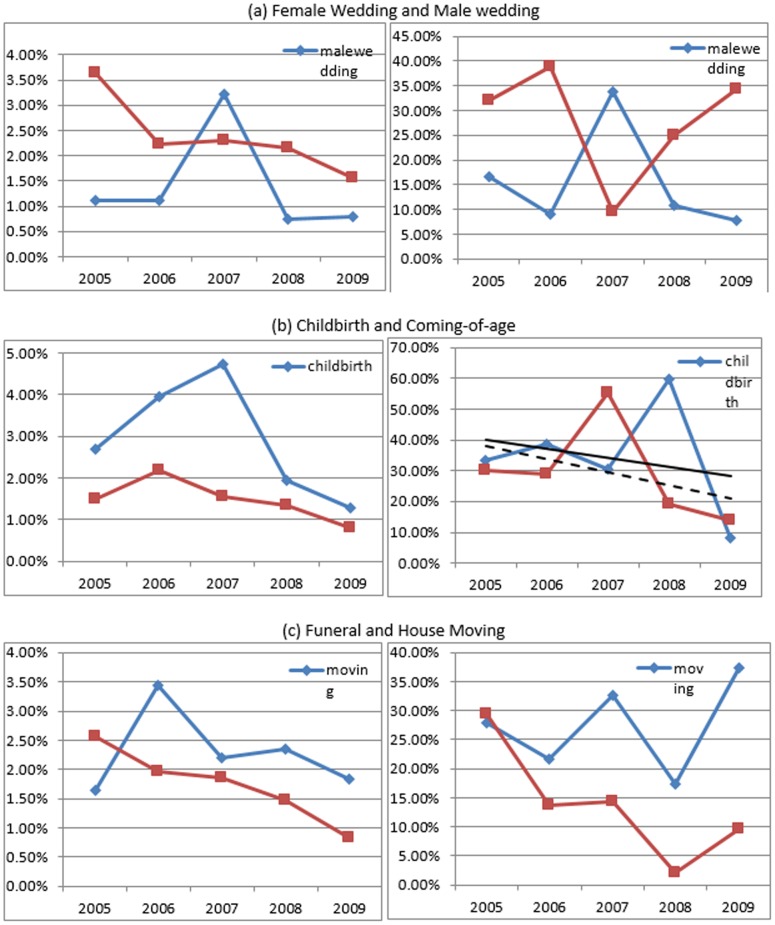
Gift Network Centrality by Occasion. *Source:* Author's social network data. *Notes:* Left Figures show normalized out-degree network centrality (influence), while right Figures show normalized in-degree network centrality (popularity).

First, male wedding is one of the most publicly participated social occasions. The size of wedding ceremonies signals wealth to fellow residents. However, when females get married, only closest relatives, friends and neighbors attend. Brides' families have little motive to show wealth. The contrasting pattern between female and male wedding networks (Figure 5a versus Figure 5b) illustrates that the centralities for male wedding networks are smaller than female wedding (Figure 6a), meaning that people more widely exchange gifts during male weddings. One main reason might be that unbalanced sex ratio triggers marriage market competition favoring brides. Groom's families have to throw bigger parties and invite more guests to signal to bride's families [5]. Another point might be that the patrilineal society with son preference makes wedding ceremony mainly an event for grooms' families with the purpose of extending network and achieving higher social status [36].

Second, childbirth is more of a private ceremony, in which only the closest relatives and friends are invited. Coming-of-age, a ceremony solely for sons, is more formally and widely held [8]. A ritual is usually hosted followed by a large banquet, signaling the growing up of a son to the fellow residents as well as the potential matchmakers. The contrasting pattern between childbirth network (Figure 5c) and coming-of-age network (Figure 5d) demonstrates smaller centralities and wider participation for coming-of-age ceremony (Figure 6b).

A third comparison is between house-moving and funeral. Most fellow residents attend funerals, which last for a few days and require much assistance, while fewer people attend house-moving ceremonies. Moreover, funerals signal social status [5], [30]. The contrasting pattern is verified by a comparison of network map between the two occasions (Figure 5e and Figure 5f). Figure 6c further suggests that funerals are more widely attended as the centralities are significantly smaller than house-moving.

Comparing six major occasions over recent years, funeral, coming-of-age and male wedding are the most widely held, while female wedding and child birth are the least widely celebrated. Meanwhile, people exchange gifts more widely in coming-of-age ceremony and male wedding compared to female wedding, house moving and child birth. The gift-sending network centralities decline for nearly all ceremonies, especially for female wedding and funeral, while gift-receiving network centralities decline the most for funeral and childbirth.

### Gift Exchanges, Mutual Help and Informal Insurance

Mutual help and informal risk-pooling are probably the most fundamental motives behind gift exchanges. With the constrained access to financial resources in developing contexts, gift exchanges may help pool available resources together to cover lumpy ceremony expenditures (Table 3), smooth consumption and facilitate productive investment, mitigating idiosyncratic shocks to impoverished families. Along this line, our exploration of risk-sharing motives and their efficiency in gift networks is underway but out of the scope of this paper.

Gift exchanges intensify connections among households, which may also mobilize resources pooling in other important dimensions. Table S4 summarizes major forms of mutual assistance in our surveyed communities. Exchanging labor during busy seasons and house building is more often than other forms of mutual help. To compensate for labor cost, hosts tended to prepare food for the exchanged labor, but now they are prone to hire labor in the market with cash wage, especially for villages with good market access. Nonetheless, Table 5 shows significant correlations between centrality in the networks and frequency of labor exchange, job information exchange and elderly/child care. Meanwhile, households that spend more on gifts do not necessarily engage more in these informal insurance arrangements.

**Table 5:** Correlation between Gift-giving Engagement and Resources Exchange.

Resources Exchange Network Engagement	Labor exchange (busy season)	Labor exchange (house building)	Job info exchange	Production tool exchange	Elderly/child care
Gift expenditure per capita	0.050	−0.030	−0.070	0.050	0.002
Bonacich Centrality	−0.127**	−0.158***	−0.116**	0.007	−0.121**

*Source:* calculation based on Authors' survey data.

*Notes:* [1] Each cell presents pairwise correlation coefficient with its significance.

[2] All five columns indicating resources exchange set the possible values into five categories: 1 = most often, 2 = very often, 3 = somewhat often, 4 = not at all often, 5 = rare or never.

Table S5 shows that more than half of the rural residents surveyed are in debt, and people generally rely on relatives when faced with cash shortage. Most loans do not carry an interest, especially those offered by relatives and neighbors. Table 6 indicates that households with higher centrality and those spend more on gifts are associated with more debt accumulation, and significantly more loans are granted by relatives. Meanwhile, they are more likely to ask relatives/friends/neighbors for help when faced with cash shortage. Surprisingly, households more central to the gift networks significantly resort to selling blood when faced with cash shortage. This suggests that selling blood might be complementary to other means in coping with cash shortage, and higher gift network centrality may be achieved at the cost of higher probability of selling blood [35].

**Table 6:** Correlation between Gift-giving Engagement and Informal Credit.

Credit Insurance Network Engagement	Debt (end of 2009)	Debt from relatives	Debt from neighbors	Interest rate	Cash shortage coping: relatives/friends/neighbors	Cash shortage coping: blood sales
Gift expenditure per capita	0.232***	0.143***	0.053	−0.038	0.115**	−0.088
Bonacich Centrality	0.132**	0.180***	0.110*	−0.112	0.122**	0.160***

*Source:* calculation based on Authors' survey data.

*Notes:* Each cell presents pairwise correlation coefficient with its significance.

### How is Gift Network Structure Determined?

The network structure depicted in Figure 1 is built upon all gift exchanges. Each group member plays a role in its formation. Having discussed potential factors motivating gift-giving, we are curious about the determinants of the network structure. We first measure individual *out-degree centrality* and *in-degree centrality* that only account for direct links. The out-degree (in-degree) centrality aggregates all direct gift links an individual send to (receive from) others. The two measures gauge very different dimensions of network engagement. Individuals of higher *in-degree centrality* tend to be more popular in the network, while those of higher out-degree centrality tend to exert more influence. Moreover, an individual with fewer number of direct links may not be isolated at all as an indirect link with a central agent in the network may mean much more than a handful of direct links with less central agents. Thus, we also measure two centrality indicators that consider both direct and indirect ties, i.e. *Bonacich Centrality* [3] and *Closeness centrality*. *Bonacich Centrality* makes use of all direct *and* indirect connections of the actors in one's neighborhood. The more connections the actors in one's neighborhood have, the more central one is. However, *Closeness centrality* solely gauges the shortest distance of the respondent to all others, both directly *and* indirectly linked, in the network. Therefore, these four most often used measures complement each other in capturing network features from distinct dimensions (Table S3).

Table 7 examines potential correlates of network structure, including main demographic and socioeconomic characteristics (Table 1), using the four centrality measures. Built on the conventional network centrality calculations that make use of the existence of pairwise links, each gift link is weighed by its monetary value and normalized network centralities are calculated using the network analysis software UCINET version 6. We treat gift exchanges each year as a complete network in calculating network centralities and take advantage of the longitudinal data structure to estimate household fixed effect models. Unobservable time-invariant characteristics, including potential confounded factors, are cancelled out, so are the observable time-invariant characteristics. Three main findings follow.

**Table 7:** Socioeconomic Determinants of Network Structure (Household Fixed Effects Model).

	R1	R2	R3	R4
	log Bonacich Centrality	log In-degree Centrality (Popularity)	log Out-degree Centrality (Influence)	log Closeness Centrality
Ceremony organizer (1 = yes, 0 = no)	0.44	2.03***	0.09	−0.02
	(0.59)	(0.13)	(0.18)	(0.01)
Per capita income (log)	0.40**	0.06	0.01	0.01*
	(0.17)	(0.04)	(0.05)	(0.00)
Log (blood relatives' Bonacich network centrality)	6.64*	1.82**	1.00	0.02
	(3.85)	(0.82)	(1.19)	(0.01)
Marital status (1 = married, 0 = no)	0.74***	0.02	0.01	0.01
	(0.26)	(0.11)	(0.11)	(0.01)
Share of unmarried youth	1.05	0.06	0.32	0.11***
	(0.83)	(0.18)	(0.26)	(0.02)
Share of the elderly	0.72	−0.07	−0.07	−0.07***
	(0.92)	(0.20)	(0.29)	(0.02)
age	0.08*	0.00	0.00	0.00
	(0.04)	(0.01)	(0.01)	(0.00)
Household size	−0.04	0.00	−0.05	0.00
	(0.14)	(0.03)	(0.04)	(0.00)
Gini coefficient (between 0 and 1)	15.80***	0.35	6.88***	0.04
	(2.99)	(0.64)	(0.93)	(0.06)
Machine (#)	−0.07	0.06	−0.66	−0.04
	(1.36)	(0.29)	(0.42)	(0.03)
Land size (mu)	0.04	0.00	0.05***	0.00
	(0.06)	(0.01)	(0.02)	(0.00)
Cow (#)	0.33	0.06	0.06	0.03***
	(0.25)	(0.05)	(0.08)	(0.01)
Horse (#)	0.22	−0.12	−0.37	0.06***
	(0.72)	(0.16)	(0.23)	(0.02)
# large diseases in the past 2 years	−0.63*	0.13	−0.28**	−0.05***
	(0.38)	(0.08)	(0.12)	(0.01)
# livestock death in the past 2 years	0.16	0.10	−0.05	−0.04***
	(0.44)	(0.09)	(0.13)	(0.01)
r2	0.09	0.10	0.21	0.16
N	552	552	552	552

*Notes:* Standard errors reported in the parentheses. All household network centrality indicators are normalized. Year fixed effects are controlled. All four columns treat gift exchanges in each year as a complete network when calculating centralities. The data on measured centralities are merged with three-wave household survey. Refer to Table S3 for details about network structure measures used here.

First, Family economic conditions determine network centralities. Families with higher income tend to have higher network centralities, especially those measures accounting for both direct and indirect ties. Specifically, a 10 percent increase in per capita income raises Bonacich centrality and closeness centrality by 4 percent and .1 percent, respectively. Families with more productive assets, such as farm land, cows and horses, are associated with higher closeness centrality or out-degree centrality. For example, one additional cow in a family leads to 3 percent higher closeness centrality, while one more horse is associated with 6 percent higher closeness centrality. Families experiencing big diseases or loss of livestock tend to have lower centralities, especially closeness centrality and out-degree centrality. However, these families often receive help from others, which promotes in-degree centrality. One more family members suffered from big diseases in the last two years reduces closeness centrality and out-degree centrality by 5 percent and 28 percent, respectively. Therefore, the negative impact of human diseases and livestock deaths and the positive effect of productive assets on closeness centrality are similar in size.

Second, family demographic characteristics affect network centralities. Larger blood relatives' network centrality predict higher centralities in gift networks, especially in-degree and Bonacich centrality. A 10 percent increase in the Bonacich centrality of relatives' networks rises Bonacich centrality and in-degree centrality of gift networks by 66 percent and 18 percent, respectively. Household with more unmarried youth or fewer senior members tend to have significantly higher closeness centrality. Given household size, a 10 percent higher share of unmarried youth increases closeness centrality by 1.1 percent, while the same reduction in the share of older people rises closeness centrality by .7 percent. Families with married heads in older age are associated with higher centralities, especially Bonacich centrality. As expected, households hosted ceremonies before have higher in-degree centrality as they are more likely to receive gifts, while their out-degree centrality is not necessarily larger.

Third, families residing in more unequal communities have higher out-degree centrality and Bonacich centrality. Specifically, one percentage point increase in Gini coefficient respectively promotes Bonacich centrality and out-degree centrality by 15.8 percent and 6.9 percent. Combined with the positive association between unmarried youth and network centralities, these findings are in line with the hypothesis of concern for relative standing. High income inequality may push up the price of gift exchanges among the rich, while families of lower economic status have to keep up with the Joneses. Meanwhile, living in the Chinese communities with skewed sex ratios that favor females, families with unmarried youth may be motivated to signal to the competitive marriage market by spending more on gifts.

Lastly, though the four measures of network centrality incorporate distinctive information on network structure, income and economic inequality, health shocks, having unmarried youth, and relatives' network centrality are more salient and more consistent predictors among the observables in our multi-wave survey data.

## Conclusions

The tradition of keeping written gift records received from celebrating household social events offers researchers a great potential to collect valuable social network datasets in many countries. This paper draws researchers' attention to a number of unique features of this widely available but much underutilized data source and discusses how gift records well fit social network studies at the individual level and the dyadic link level.

We document the gift-giving trend in rural China. We argue that the observed fast growing gift-giving may not be due to the social exclusion motive. Rather, the evidence points to concern for relative standing and booming windfall and other non-earned income to village residents. While mutual help and informal risk-pooling may not be the cause of gift spending escalation, it correlates intensely with one's engagement in the gift networks. In the meantime, blood relatives' network size, marriage market pressure, income and economic inequality are found to be among the major gift network structure determinants.

Though collecting gift record data may hardly be general enough to follow in some contexts, our insights may help future studies to overcome some of the key issues. Moreover, this paper may help improve our understanding of networks, especially in underdeveloped contexts. Future research projects are underway, which involves deriving spatial instruments from the gift network structure to identify peer effect in social behavior, exploring determinants of gift spending escalation, and evaluating its impact on individual well-being.

## Supporting Information

Table S1 **Income Mobility of the Surveyed Villages (Transition Matrix, 2004–2006).** *Source:* Author's household survey data. *Notes:* Shorrocks' MET - the Prais index: **0.913** (SE: .02064661; CI: [0.872, 0.953]). Atkinson et al. Mobility Ratio: **0.389** (SE: .02293795; CI: [0.344, 0.434]). The rows denote income quartiles in the initial period, while the columns denote income quartiles in the later period.
(DOCX)

Table S2 **Income Mobility of the Surveyed Villages (Transition Matrix, 2006–2009).** *Source:* Author's household survey data. *Notes:* Shorrocks' MET - the Prais index: **0.823** (SE: .02649932; CI: [0.771, 0.875]). Atkinson et al. Mobility Ratio: **0.326** (SE: .0276683; CI: [0.272, 0.380]). The rows denote income quartiles in the initial period, while the columns denote income quartiles in the later period.
(DOCX)

Table S3 **Definition and Measures of Social Network Structure/Engagement.** *Notes:* The centrality of an individual in a network captures the idea of power and prominence in a certain social structure. I assume the network *g* has n individuals. Comparing among network structure measures, the degree centrality and closeness centrality are equal for two extreme cases, star network and cycle network, while they are valued differently in this range. The major shortcoming for the two centrality measures is that it excludes the case when actions of a person influence actions of their neighbors which in turn feedback on the initiator. The degree centrality only takes into account the immediate ties each node has. An individual might be centrally tied to a large number of others, but those others are disconnected from the network as a whole. The closeness centrality solely depends on the length of the shortest paths between nodes in network, while it is possible that ties are not perfectly reliable and other paths of different lengths may take effects. Both direct and indirect influences in a network are captured by Bonacich centrality. Compared to other two centrality measures, only Bonacich centrality is parameter-free. Bonacich centrality has behavior foundation that is derived from Nash equilibrium of a non-cooperative game, while other centrality measures are mainly geometric in nature. Bonacich centrality can be derived using the framework of a linear interaction of behaviors among peers where individual behavior is a weighted average of peers' behavior.
(DOCX)

Table S4 **Labor/Information/Production Tool Exchanges among Neighbor/Friends/Relatives.** *Source:* Author's household survey data.
(DOCX)

Table S5 **Household Debt and Credit (End of 2009).** *Source:* Author's household survey data. *Notes:* “Major sources of debt (%)” categories stock value at the end of 2009, while “Major ways to deal with credit constraints” categories flow value during 2009. Meanwhile, the latter only includes households faced with credit constraints, which accounts for 90.1% of the surveyed households.
(DOCX)
