# Supplementary material for: Gift-Giving and Network Structure in Rural China: Utilizing Long-Term Spontaneous Gift Records
Source: PLoS One. 2014 Aug 11;9(8):e102104. doi: 10.1371/journal.pone.0102104 (PMC4128647; doi:10.1371/journal.pone.0102104)
Supplement: Table S1 — Income Mobility of the Surveyed Villages (Transition Matrix, 2004–2006). Source: Author's household survey data. Notes: Shorrocks' MET - the Prais index: 0.913 (SE: .02064661; CI: [0.872, 0.953]). Atkinson et al. Mobility Ratio: 0.389 (SE: .02293795; CI: [0.344, 0.434]). The rows denote income quartiles in the initial period, while the columns denote income quartiles in the later period. (DOCX) [file pone.0102104.s001.docx]

**Table S1 Income Mobility of the Surveyed Villages (Transition Matrix, 2004 – 2006)**

| t t+1 | Lowest 20% | Lower 20% | Mid 20% | Higher 20% | Highest 20% |
| --- | --- | --- | --- | --- | --- |
| Lowest 20% | 0.27 | 0.24 | 0.26 | 0.13 | 0.11 |
| Lower 20% | 0.21 | 0.27 | 0.19 | 0.20 | 0.13 |
| Mid 20% | 0.22 | 0.24 | 0.18 | 0.21 | 0.16 |
| Higher 20% | 0.17 | 0.18 | 0.23 | 0.22 | 0.20 |
| Highest 20% | 0.13 | 0.11 | 0.14 | 0.20 | 0.42 |

*Source:* Author’s household survey data.

*Notes:* Shorrocks' MET - the Prais index: **0.913** (SE: .02064661; CI: [0.872 , 0.953])

Atkinson et al. Mobility Ratio: **0.389** (SE: .02293795; CI: [0.344 , 0.434])

The rows denote income quartiles in the initial period, while the columns denote income quartiles in the later period.
