# Supplementary material for: Gift-Giving and Network Structure in Rural China: Utilizing Long-Term Spontaneous Gift Records
Source: PLoS One. 2014 Aug 11;9(8):e102104. doi: 10.1371/journal.pone.0102104 (PMC4128647; doi:10.1371/journal.pone.0102104)
Supplement: Table S2 — Income Mobility of the Surveyed Villages (Transition Matrix, 2006–2009). Source: Author's household survey data. Notes: Shorrocks' MET - the Prais index: 0.823 (SE: .02649932; CI: [0.771, 0.875]). Atkinson et al. Mobility Ratio: 0.326 (SE: .0276683; CI: [0.272, 0.380]). The rows denote income quartiles in the initial period, while the columns denote income quartiles in the later period. (DOCX) [file pone.0102104.s002.docx]

**Table S2 Income Mobility of the Surveyed Villages (Transition Matrix, 2006 – 2009)**

| t t+1 | Lowest 20% | Lower 20% | Mid 20% | Higher 20% | Highest 20% |
| --- | --- | --- | --- | --- | --- |
| Lowest 20% | 0.35 | 0.21 | 0.24 | 0.13 | 0.07 |
| Lower 20% | 0.18 | 0.35 | 0.21 | 0.16 | 0.10 |
| Mid 20% | 0.17 | 0.15 | 0.34 | 0.23 | 0.11 |
| Higher 20% | 0.11 | 0.14 | 0.23 | 0.27 | 0.25 |
| Highest 20% | 0.10 | 0.16 | 0.14 | 0.20 | 0.40 |

*Source:* Author’s household survey data.

*Notes:* Shorrocks' MET - the Prais index: **0.823** (SE: .02649932; CI: [0.771 , 0.875])

Atkinson et al. Mobility Ratio: **0.326** (SE: .0276683; CI: [0.272 , 0.380])

The rows denote income quartiles in the initial period, while the columns denote income quartiles in the later period.
