# Supplementary material for: Gift-Giving and Network Structure in Rural China: Utilizing Long-Term Spontaneous Gift Records
Source: PLoS One. 2014 Aug 11;9(8):e102104. doi: 10.1371/journal.pone.0102104 (PMC4128647; doi:10.1371/journal.pone.0102104)
Supplement: Table S3 — Definition and Measures of Social Network Structure/Engagement. Notes: The centrality of an individual in a network captures the idea of power and prominence in a certain social structure. I assume the network g has n individuals. Comparing among network structure measures, the degree centrality and closeness centrality are equal for two extreme cases, star network and cycle network, while they are valued differently in this range. The major shortcoming for the two centrality measures is that it excludes the case when actions of a person influence actions of their neighbors which in turn feedback on the initiator. The degree centrality only takes into account the immediate ties each node has. An individual might be centrally tied to a large number of others, but those others are disconnected from the network as a whole. The closeness centrality solely depends on the length of the shortest paths between nodes in network, while it is possible that ties are not perfectly reliable and other paths of different lengths may take effects. Both direct and indirect influences in a network are captured by Bonacich centrality. Compared to other two centrality measures, only Bonacich centrality is parameter-free. Bonacich centrality has behavior foundation that is derived from Nash equilibrium of a non-cooperative game, while other centrality measures are mainly geometric in nature. Bonacich centrality can be derived using the framework of a linear interaction of behaviors among peers where individual behavior is a weighted average of peers' behavior. (DOCX) [file pone.0102104.s003.docx]

**Table S3 Definition and Measures of Social Network Structure/Engagement**

| Indicators | Definition | Calculation |
| --- | --- | --- |
| In-degree Centrality  (popularity) | measures number (intensity) of links the respondent receives from peers | Known as in-degree centrality, $g_{ij}$is a binary (continuous) variable denoting (intensity of) the link from j to i. $C_{d}(i;g)=\sum_{j=1}^{n} g_{ij}/(n-1)$ |
| Out-degree Centrality  (Influence) | measures number (intensity) of links the respondent sends out to peers | Known as out-degree centrality, $g_{ij}$is a binary (continuous) variable denoting (intensity of) the link from i to j $C_{d}(i;g)=\sum_{j=1}^{n} g_{ij}/(n-1)$ |
| Closeness  Centrality | distance of the respondent to all others in the network | $\sum_{j\neq i} d(i,j;g)$ denotes the geodesic distance from i to all other n-1 individuals in network $g$. $C_{c}(i;g)=\frac{n-1}{\sum_{j\neq i} d(i,j;g)}$ |
| Bonacich Centrality | Respondent’s centrality, weighted by the centrality of those to whom he or she sends ties | $g_{ij}^{k}$ is the amount of walks of length k that exist between i and j in network $g$. $\alpha\sigma$is decaying parameter for indirect ties $C_{b}(i;g,\alpha\sigma)=\sum_{j=1}^{n} \sum_{k=0}^{\infty} (\alpha\sigma)^{k}g_{ij}^{k}$ |

*Notes:* The centrality of an individual in a network captures the idea of power and prominence in a certain social structure. I assume the network $g$ has n individuals. Comparing among network structure measures, the degree centrality and closeness centrality are equal for two extreme cases, star network and cycle network, while they are valued differently in this range. The major shortcoming for the two centrality measures is that it excludes the case when actions of a person influence actions of their neighbors which in turn feedback on the initiator. The degree centrality only takes into account the immediate ties each node has. An individual might be centrally tied to a large number of others, but those others are disconnected from the network as a whole. The closeness centrality solely depends on the length of the shortest paths between nodes in network, while it is possible that ties are not perfectly reliable and other paths of different lengths may take effects. Both direct and indirect influences in a network are captured by Bonacich centrality. Compared to other two centrality measures, only Bonacich centrality is parameter-free. Bonacich centrality has behavior foundation that is derived from Nash equilibrium of a non-cooperative game, while other centrality measures are mainly geometric in nature. Bonacich centrality can be derived using the framework of a linear interaction of behaviors among peers where individual behavior is a weighted average of peers’ behavior.
