# Supplementary material for: Gift-Giving and Network Structure in Rural China: Utilizing Long-Term Spontaneous Gift Records
Source: PLoS One. 2014 Aug 11;9(8):e102104. doi: 10.1371/journal.pone.0102104 (PMC4128647; doi:10.1371/journal.pone.0102104)
Supplement: Table S4 — Labor/Information/Production Tool Exchanges among Neighbor/Friends/Relatives. Source: Author's household survey data. (DOCX) [file pone.0102104.s004.docx]

**Table S4 Labor/Information/Production Tool Exchanges among Neighbor/Friends/Relatives**

| Categories | | Labor exchange (busy season) | Labor exchange (house building) | Job info  exchange | Production tool exchange | Elderly/kids  care |
| --- | --- | --- | --- | --- | --- | --- |
| Most often | | 5.9% | 6.2% | 3.9% | 4.7% | 1.8% |
| Very often | | 14.8% | 19.5% | 11.2% | 13.9% | 11.2% |
| Somewhat often | | 12.1% | 15.4% | 11.9% | 14.7% | 12.9% |
| Not at all often | | 12.4% | 11.5% | 11.3% | 12.4% | 15.9% |
| Rare or never | | 54.8% | 47.5% | 61.7% | 54.4% | 58.3% |
| Days in total | Median | 0 | 0 | - | - | - |
|  | p75 | 5 | 5 | - | - | - |
|  | Mean | 3.62 | 4.86 | - | - | - |
|  | Std. Dev. | 7.4 | 15.6 | - | - | - |

*Source:* Author’s household survey data.
