# Supplementary material for: Gift-Giving and Network Structure in Rural China: Utilizing Long-Term Spontaneous Gift Records
Source: PLoS One. 2014 Aug 11;9(8):e102104. doi: 10.1371/journal.pone.0102104 (PMC4128647; doi:10.1371/journal.pone.0102104)
Supplement: Table S5 — Household Debt and Credit (End of 2009). Source: Author's household survey data. Notes: “Major sources of debt (%)” categories stock value at the end of 2009, while “Major ways to deal with credit constraints” categories flow value during 2009. Meanwhile, the latter only includes households faced with credit constraints, which accounts for 90.1% of the surveyed households. (DOCX) [file pone.0102104.s005.docx]

**Table S5 Household Debt and Credit (end of 2009)**

| Percentage of families with debt (%) | 57.1 |
| --- | --- |
| Percentage of families grant a loan (%) | 6.0 |
| Percentage of families with debt & grant a loan (%) | 3.1 |
| The median amount of debt (RMB) when incurred | 6,000 |
| The median amount of loan (RMB) when granted | 5,000 |
| Major sources of debt (%) |  |
| *Relatives* | 70.9 |
| *Neighbors* | 4.0 |
| *Rural credit union* | 25.1 |
| *Usury* | 8.6 |
| *Others (e.g. non-local friends, fertilizer retailers)* | 1.8 |
| When borrowing from relatives |  |
| *Percentage of zero interest* | 89.8 |
| *Median monthly interest rate (%) when charging interest* | 1.0 |
| When borrowing from neighbors |  |
| *Percentage of zero interest* | 70.0 |
| *Median monthly interest rate (%) when charging interest* | 2.0 |
| When borrowing from formal rural credit union |  |
| *Median monthly interest rate (%)* | 1.0 |
| When borrowing from usury lenders |  |
| *Median monthly interest rate (%)* | 3.0 |
| When borrowing from others |  |
| *Median monthly interest rate (%)* | 0.0 |
| Major ways to deal with credit constraints in 2009 (%) |  |
| *Borrowing from relatives, neighbors and friends* | 45.3 |
| *Selling assets* | 40.6 |
| *Working out or receiving remittance* | 26.1 |
| *Applying for a loan* | 7.4 |
| *Borrowing from usury lenders* | 2.0 |

*Source:* Author’s household survey data.

*Notes:* “Major sources of debt (%)” categories stock value at the end of 2009, while “Major ways to deal with credit constraints” categories flow value during 2009. Meanwhile, the latter only includes households faced with credit constraints, which accounts for 90.1% of the surveyed households.
